# Novel Techniques to Measure the Sensory, Emotional, and Physiological Responses of Consumers toward Foods

**DOI:** 10.3390/foods10112620

**Published:** 2021-10-29

**Authors:** Damir D. Torrico

**Affiliations:** Department of Wine, Food and Molecular Biosciences, Faculty of Agriculture and Life Sciences, Lincoln University, Lincoln 7647, New Zealand; Damir.Torrico@lincoln.ac.nz; Tel.: +64-3-423-0641

Sensory science is an evolving field that has been incorporating technologies from different disciplines. Sensory evaluation is very important for the food and beverage industries as it can provide applied insights regarding the reactions of consumers towards products. Traditional techniques such as discrimination, descriptive and affective tests have been utilized for understanding the sensory properties of foods and evaluating the hedonic and emotional responses of consumers. However, these techniques have their drawbacks and sensory scientists have been working on improving these methodologies or developing new measurement systems. For instance, sensory scientists have been investigating alternative measurements of consumers by using physiological responses. On the other hand, research has been conducted to understand the effects of context in the consumer evaluation of foods and beverages. Moreover, techniques such as home-use tests or virtual reality evaluations are being regarded as having more ecological validity since they require participants to taste the products in their day-to-day environments. This Special Issue of *Foods* aimed to understand the development of novel techniques to measure the sensory, emotional, and physiological responses of consumers toward food.

Traditional sensory methods use self-reported responses to gather information from trained panels or untrained consumers. However, these techniques have the limitation of not capturing the totality of responses from these participants [1]. Moreover, self-reported responses in sensory tests are exposed to cognitive biases since they are not generated immediately during decision making [2]. A complementary measurement of self-reported responses is the incorporation of physiological reactions that can be achieved by the use of biometrics. Responses such as heart rate, body temperature, and facial expressions have been shown to tap into the unconscious responses of consumers in sensory tests. In a study that evaluated energy drinks, Mehta, Sharma, Kanala, Thakur, Harrison and Torrico [1] investigated the self-reported responses (including hedonic and emotional) and facial expressions to determine the degree to which participants liked these products. They concluded that participants elicited more self-reported positive than negative emotions for the energy drinks, and that the implicit emotional responses through facial expressions were important in the discrimination of the products. In a study of emotions toward beer products, Gonzalez Viejo et al. [3] concluded that sweet beers were associated with higher liking and positive emotions; on the other hand, bitter beers were associated with negative self-reported emotions and negative feelings expressed through facial expressions and other physiological measurements.

These previous studies showed the importance of assessing biometric responses when testing for familiar products. However, consumers nowadays are exposed to a wide variety of foods from different countries and production systems. An important food trend that is influencing consumers’ purchasing decisions is the sustainability of foods/beverages production systems. The alternative protein movement has been pushing food developers to reformulate existing products with new ingredients and at the same time to keep the acceptability of those products at similar or higher levels. In this context, understanding the consumer assessments of emerging ingredients such as plant- and insect-based products is becoming crucial for companies that are implementing alternative protein foods. In this regard, Gupta et al. [4] evaluated plant-based yogurt products using hedonic, emotional (check-all-that-apply methodology with emotional terms and emojis), and physiological responses of consumers. They found that the use of emojis was effective in the characterization of cross-cultural preferences of different yogurt formulations compared to those of hedonic ratings and facial expressions. In another study, Fuentes, Wong and Gonzalez Viejo [2] evaluated the sensory and biometric responses of consumers toward insect-based food snacks. They concluded that snacks containing visible insects in their presentations had a lower degree of acceptance and negative emotional responses compared to snacks containing non-visible insects or no insects in their formulations. This study demonstrated that the appearance of products could have a profound effect on the hedonic and emotional assessments of foods containing insects.

Consumers’ purchasing decisions are not only affected by the intrinsic sensory properties of the products but also by the extrinsic information that is provided at the time of purchasing. De Wijk et al. [5] evaluated the effects of familiarity and branding on the sensory experiences of soy sauces using facial expressions and video-based heart rate measurements. They found that liking and arousal toward the products were affected by taste but not by branding and familiarity. On the other hand, biometric measurements (facial expressions and heart rate) were affected by the branding and familiarity of the soy sauces. This study also showed that the assessment of facial expressions and heart rate can be done remotely without the use of centrally located laboratories, and only by using image and video recording devices. Another important parameter to measure the implicit (unconscious) reactions of consumers is the Approach Avoidance Task (AAT). Humans, in general, tend to avoid (leaning backwards) the stimuli that they dislike, and they tend to approach (leaning forward) the stimuli that they like when they are doing a sensory test. Brouwer et al. [6] evaluated the AAT using a mobile version that consumers can use at their homes using their phones to measure their reactions (AAT and valance ratings) toward pictures of palatable and unpalatable foods. They found that the ATT measurements obtained by this mobile version could complement the rating scores of participants, which can be useful in understanding the intrinsic reactions of consumers.

Traditional consumer tests focused on understanding the links between foods and consumers. Measuring the hedonic responses and preferences is very important for companies when launching new products into the marketplace. Traditional consumer tests use booths to isolate participants from external factors (noises, smells). This is a common practice in sensory evaluation since consumers can be easily distracted from the sensory assessment and their responses can be biased by these external factors. However, isolated booths might not represent the “real” environments where consumers usually taste their products. In some cases, the combinations of environment and product can enhance the sensory experiences of consumers. For that reason, new research has been done to understand the effect of environments on sensory perception. Montero et al. [7] studied the consumer acceptance of ready-to-eat (RTE) meals during storage using a home-use test (HUT). They concluded that the HUT methodology can enhance the sensory experience of consumers when tasting the products.

In some cases, HUT might not be possible to conduct due to budget constraints or the requirement of specific preparations of the products. For those factors, researchers have started to use alternative methods to measure the effect of the environment on the consumer experience. New technologies such as virtual and augmented reality are becoming very useful when testing for the environment. Crofton et al. [8] explored the effects of immersive virtual reality (VR) technologies on the sensory perception of beef and chocolate. They found that VR had a significant effect on participants’ hedonic responses to the products and that consumers’ level of engagement was different depending on the context. In a similar study, Kong et al. [9] demonstrated that VR had slight effects on the emotions of consumers towards chocolate products. In a study of wines, Torrico et al. [10] found that VR environments, in general, affected the perception of floral aroma and emotions.

In conclusion, this Special Issue showed that sensory evaluation is changing from the sole use of rating/ranking scales to the incorporation of biometric measurements and the use of immersive technologies. The development of new sensory techniques is an ongoing task, which is constantly looking for accurate measurements of consumers’ responses. The editor hopes that the findings from this Special Issue can help the broader scientific community to understand the use of novel sensory science techniques that can be used in the evaluation of food and beverage products.
